# Comparison of Variable-Number Tandem-Repeat Markers typing and IS*1245 *Restriction Fragment Length Polymorphism fingerprinting of *Mycobacterium avium *subsp. *hominissuis *from human and porcine origins

**DOI:** 10.1186/1751-0147-52-21

**Published:** 2010-03-10

**Authors:** Taneli Tirkkonen, Jaakko Pakarinen, Elina Rintala, Terhi Ali-Vehmas, Harri Marttila, Olli AT Peltoniemi, Johanna Mäkinen

**Affiliations:** 1 Faculty of Veterinary Medicine, Department of Production Animal Medicine, POB 66, FIN 00014, University of Helsinki, Finland; 2 A-Farmers Ltd, POB 910, FIN 60061 Atria, Finland; 3 Faculty of Agriculture and Forestry, Department of Applied Chemistry and Microbiology, University of Helsinki, Finland; 4 Faculty of Veterinary Medicine, Department of Basic Veterinary Sciences, POB 56, FIN 00014 University of Helsinki, Finland; 5 Mycobacterial Reference Laboratory, The National Institute for Health and Welfare, Kiinanmyllynkatu 13, FIN 20520, Turku, Finland; 6 Lieto municipality, Po box 24, FIN 21421 Lieto, Finland

## Abstract

**Background:**

Animal mycobacterioses are regarded as a potential zoonotic risk and cause economical losses world wide. *M. avium *subsp. *hominissuis *is a slow-growing subspecies found in mycobacterial infected humans and pigs and therefore rapid and discriminatory typing methods are needed for epidemiological studies. The genetic similarity of *M. avium *subsp. *hominissuis *from human and porcine origins using two different typing methods have not been studied earlier. The objective of this study was to compare the IS*1245 *RFLP pattern and MIRU-VNTR typing to study the genetic relatedness of *M. avium *strains isolated from slaughter pigs and humans in Finland with regard to public health aspects.

**Methods:**

A novel PCR-based genotyping method, variable number tandem repeat (VNTR) typing of eight mycobacterial interspersed repetitive units (MIRUs), was evaluated for its ability to characterize Finnish *Mycobacterium avium *subsp. *hominissuis *strains isolated from pigs (n = 16) and humans (n = 13) and the results were compared with those obtained by the conventional IS*1245 *RFLP method.

**Results:**

The MIRU-VNTR results showed a discriminatory index (DI) of 0,92 and the IS*1245 *RFLP resulted in DI 0,98. The combined DI for both methods was 0,98. The MIRU-VNTR test has the advantages of being simple, reproducible, non-subjective, which makes it suitable for large-scale screening of *M. avium *strains.

**Conclusions:**

Both typing methods demonstrated a high degree of similarity between the strains of human and porcine origin. The parallel application of the methods adds epidemiological value to the comparison of the strains and their origins. The present approach and results support the hypothesis that there is a common source of *M. avium *subsp. *hominissuis *infection for pigs and humans or alternatively one species may be the infective source to the other.

## Background

The bacteria belonging to the *Mycobacterium avium *complex are opportunistic microorganisms ubiquitously distributed in the environment. They transmit from the environment causing a majority of atypical human and animal mycobacterial infections. The *M. avium *complex consists of closely related groups of microorganisms representing over 90% similarity at the nucleotide level, but its members differ widely in their host tropisms, microbiological characteristics, and pathogenicities. *M. avium *subsp. *hominissuis *is a common mycobacteria subspecies found in mycobacterial infected humans and pigs, whereas *M. avium *subsp. *avium *mainly infects birds [1,2].

Bacteria belonging to the *Mycobacterium avium *complex, as well as other non-tuberculous mycobacteria (NTM), are particularly infective to immunocompromized humans. Martin-Casabona et al. [3] reported 36,099 human infections by NTM, and 22,884 NTM isolates were identified to the species level in fourteen countries world wide. *M. avium *was the most common of these NTM. In Finland during 1995 to 2004, a total of 3,961 NTM isolates were obtained from human specimens and *M. avium *was reported as the most common one as it was found in 1,360 (34%) of the 3,961 cases (Finnish National Health institute, KTL, annual reports, unpublished, in Finnish).

Pig mycobacteriosis, *M. avium *being the predominant finding, is a significant problem in several European countries [4-6]. The condemnation of pork due to presumptive mycobacterial infections causes yearly losses worth approximately 0,5 million euros per the annually processed 2,2 million pig carcasses in Finland (Finnish Meat-Industry Association, annual production data 2001-2008, unpublished, in finnish). Only a small percentage of the suspected porcine mycobacterial infections are confirmed by laboratory cultivation. However, pig livers without visible lesions have been reported to contain viable mycobacteria [5]. So far, the real number of mycobacteriosis in slaughter pigs is unknown and mycobacteria contaminated pork may pass the slaughter line for human consumption or some carcasses may be rejected in vein. Control of mycobacterial infections requires knowledge of the causative agent and its epidemiology, interspecies transmission, and biodiversity within the *M. avium *strains.

The aim of this study was to compare *M. avium *subsp. *hominissuis *strains using two different typing methods to evaluate the caracteristics of these methods and to confirm the genetic similarity of the strains from human and porcine origins.

## Materials and methods

### Bacterial isolates

*M. avium *strains were isolated from slaughter pig organs (n = 16) and clinical human samples (n = 13) [1]. The isolates were identified to species level by partial sequencing of the 16S rDNA gene as described by Kirchner et al. [7]. Four strains (IWGMT 49, ATCC 15769, ATCC 25291, ATCC 35712) were included as internal standard strains for the methods used in this study.

### RFLP-typing and data analysis

The genetic typing of *M. avium *isolates by IS*1245 *Restriction Fragment Length Polymorphism (RFLP) was done as described by van Soolingen et al. [8]. A dendrogram of relatedness among the patterns was constructed by the unweighted pair group method with arithmetic averages clustering method. The RFLP clusters were defined to be 90% similarity consisting of a minimum of two strains [1].

### MIRU-VNTR typing and data analysis

MIRU-VNTR typing, data analysis and calculation of the discriminatory index were done as described by Thibault et al. [9]. The polymerase enzyme used was DynaZyme DNA polymerase (Finnzymes, Espoo, Finland). The *M. avium *subsp. *avium *ATCC 35712 strain was included into each run as a positive control to confirm the reproducibility of the MIRU-VNTR patterns. A cluster was defined as two or more isolates that were indistinguishable (100% similarity) by MIRU-VNTR (same number of tandem repeats in each loci).

## Results

### Isolation and identification of mycobacteria from human and porcine origin

Our goal was to investigate the relations between strains of mycobacteria originating from human and porcine samples. The thirteen human clinical samples and the sixteen tissue specimens from nine different pigs with presumptive tuberculous lesions were investigated for the presence of mycobacteria. All mycobacterial isolates had 16S rRNA gene sequences which were 100% identical with the 16S rRNA gene sequence of *M. avium *(GeneBank accession number CP000479) Tirkkonen et al. [1].

### MIRU-VNTR typing results

**Figure 1:**
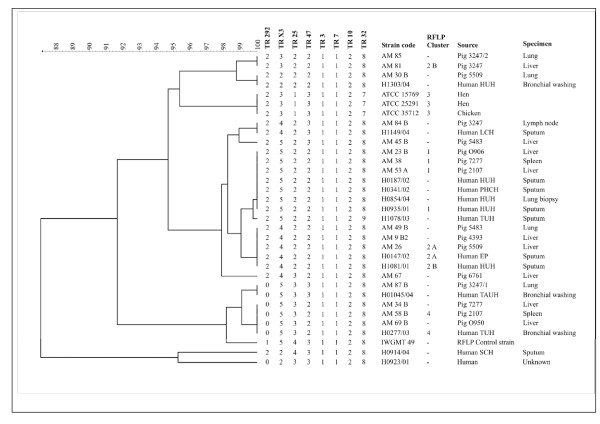
**Dendrogram of the MIRU-VNTR types of porcine, human or avian *M. avium *isolates**. The differences in MIRU-VNTR numbers were used to estimate the genetic distance. Source: OUH: Oulu University Hospital, HUH: Helsinki University Hospital, PHCH: Päijät-Häme Central Hospital, EP: Etelä-Pohjanmaa Hospital District, TAUH: Tampere University Hospital, TUH: Turku University Hospital, LCH: Lappi Central Hospital, SCH: Seinäjoki Central Hospital. IS1245 RFLP clusters are shown if the isolates clustered with RFLP. The strains were encoded and shown by numbers and letters. The -sign nominates the isolates with a unique IS1245 RFLP profile.

The genetic diversity within the mycobacterial strains of human and porcine origin was studied by MIRU-VNTR typing. The number of tandem repeats for each locus was determined and allele numbers were assigned to reflect the number of copies represented in each locus. Multilocus MIRU-VNTR types were then assigned on the basis of the combination of alleles for each locus. MIRU-VNTR differentiated the human strains into six clusters and the porcine strains into seven clusters. Most strains grouped within the common profiles. The range and mode for the different MIRUs were in TR292 (range 0-2, mode 2), in TRX3 (2-5, 5), in TR25 (1-4, 2), in TR47 (2-3, 2), in TR3 and 7(1-1, 1), in TR10 (2-2,2) and in TR32 (7-8,8). Tandem repeats were present in all strains and MIRUs studied except in MIRU 292. MIRU 292 was absent from four different porcine strains and three human strains (Figure 1). This suggests that this locus was either absent or re-arranged. However, the presence or absence of the MIRU 292 locus yielded differentiative typing information.

### Comparison of MIRU-VNTR and RFLP typing and a combination of the two methods

The MIRU-VNTR clustering of 29 *M. avium *isolates of human and porcine originh was compared with the clusters obtained by IS *1245 *RFLP analysis in an earlier study [1]. The *IS1245 *RFLP method revealed four clusters (RFLP clusters 1 to 4 in Figure 1) containing both human and porcine strains with *IS1245 *RFLP patterns ≥ 90% similar. The MIRU-VNTR typing revealed six clusters (100% similarity) containing both human and porcine isolates (Figure 1). Strains with identical MIRU-VNTR types also yielded *IS1245 *RFLP patterns ≥ 90% similar with the exception of strain AM 81 (RFLP cluster 2B). The five strains with unique MIRU-VNTR types also yielded unique *IS1245 *RFLP patterns RFLP (Figure 1). Thus, the two methods distinguished three clusters comprising of human and porcine isolates. The porcine strain AM23B and the human strain H0935/01 that differed by only one band in the IS*1245 *RFLP analysis were also identically clustered in MIRU-VNTR typing supporting the view that the strains are clonal. We conclude that the parallel application of RFLP and MIRU-VNTR typing methods amplify the confidence of the genetic relatedness between human and porcine originating *M. avium *strains.

We found one pig (3247) with four different strains (AM85 lung, AM81 liver, AM84B lymph node, AM87B lung) based on RFLP typing. The MIRU-VNTR method clustered two of these four strains together and two separately. In four pigs (5509:AM30B/AM26, 5483:AM45B/AM49B, 7277:AM38/AM34B, 2107:AM53A/AM58B) two *M. avium *isolates with different RFLP clusters were isolated from the same individual. These parallel isolates originating from the same pig clustered separately also in MIRU-VNTR. This indicates that the pigs were more often originally infected by different strains instead of mutation of one strain within the pig. Heavy environmental mycobacterial infection load could explain this phenomenon.

The similarity of RFLP patterns as well as MIRU-VNTR types between the *M. avium *subsp. *avium *reference strains was 100%. The *M. avium *subsp. *avium *isolates clustered together separately from human and porcine isolates in both used methods. The replicate DNA preparations produced identical patterns for each strain in both typing methods.

The discriminatory index (DI) for the MIRU-VNTR method was 0.92, 0,98 for the IS*1245 *RFLP method and 0,98 for the two combined. We conclude that both IS*1245 *RFLP and MIRU-VNTR methods are discriminatory, but MIRU-VNTR is less subjective and requires less labour. As a whole *M. avium *isolates from human and porcine origins showed less diversity in MIRU-VNTR method than in RFLP method. Therefore we conclude that the MIRU-VNTR clusters are probably more conserved than the IS*1245 *ones. Thus, the MIRU-VNTR analysis is more useful for longitudinal epidemiologic studies than RFLP.

## Discussion

One per ten thousand individuals in Finland is yearly infected by environmental mycobacteria, and like humans, pigs are infected by *M. avium *more often than any other NTM species [1,5]. The incidence of presumptive tuberculous lesions in slaughter pigs has increased nine-fold in Finland during the years 1998-2003, but has been decreasing since then. (The Ministry of Agriculture and Forestry of Finland and Finnish Food Safety Authority EVIRA, annual reports 1998-2008, unpublished, in finnish). Matlova et al. [10-12] suspected the bedding materials as possible infection sources for the infected pigs. Komijn et al. [5] reported isolation of *M. avium *from the mesenteric lymph nodes in of 48 out of 345 (13.9%) healthy slaughter pigs without visible tuberculous lesions in the lymph nodes. This observation suggests that visual inspection is a poor method for the detection of pig mycobacteriosis. *M. avium *isolates can be relatively resistant to heating and therefore survive in poorly heated pork products. In some cases temperature of up to 70°C is required for inhibition of *M. avium *[13]. There is justified long-term suspicion that ingestion can be a route of human *M. avium *infection [14,15]. *M. avium *strains in Finland evidence close genetic relatedness between human and porcine isolates. The results of this study are in agreement with the earlier studies reporting close genetic relatedness between human and porcine *M. avium *isolates [5,16,17]. The results also support the hypothesis there may be a common source of *M. avium *infection for pigs and humans or alternatively pigs may act as a vehicle for human infections or vice versa.

Due to the slow growth of *M. avium *on culture media, culture-independent methods are needed for the control of pathogenic mycobacteria in the meat production chain. The conserved nature of the *M. avium *genome denotes that most strain subtyping methods provide limited information on the diversity of this organism. Effective methods are needed for the detection, quantification, identification and genetic profiling of environmental mycobacteria in order to trace the environmental reservoirs of human and animal mycobacteriosis. This need is further underlined by the implication of pigs as a potential source or reservoir of human *M. avium *infection.

The most used *M. avium *typing method has been restriction fragment length polymorphism (RFLP) [18]. Komijn et al. [5] compared human and porcine *M. avium *isolates in the Netherlands by the IS*1245 *RFLP method. The RFLP patterns of 61% of the human and 59% of the porcine isolates were > 75% similar, showing close genetic relatedness. In our earlier studies, IS*1245 *RFLP patterns of 38% of the porcine and 42% of the human *M. avium *strains were > 90% similar [1].

Johansen et al [17] compared the use of IS*1311*RFLP with IS*1245*RFLP and concluded that IS*1245 *yielded higher discriminatory index, whereas IS*1311 *is easier to analyze. Due to the higher accuracy of IS*1245 *it was suitable for our research purposes. The IS*1245 *RFLP patterns of *M. avium *isolates are stable when cultured *in vitro *but less stable when passaging through live animals. The patterns may change by one or two bands over one year of laboratory cultivation [19], but in a living host the pattern may change within 69-88 days [20]. If two such multibanded patterns differ in only a few bands, it is difficult to determinate whether these patterns reflect a small variation between one strain or represent two truly different strains [21]. However, because some of our isolates differed only by one or two bands in this RFLP study they probably represent the same strains. Non identical strains in the RFLP patterns may lead to an underestimation of the epidemiological links between isolates [22,23]. RFLP is considered to be a time-consuming and technically demanding method, that requires large amounts of purified bacterial DNA and an analysis of complex banding patterns.

The use of multilocus variable tandem repeat (VNTRs) is a well established genotyping method of many pathogenic bacteria. The first bacterial species in which they were identified was *Mycobacterium tuberculosis*, being described as mycobacterial interspersed repeat units (MIRUs) [24,25]. MIRUs are mini-satellite sequences of 46-53, 58-101, and 77-101 bp in length which are distributed throughout the genome as single copies or in multiple tandem repeats [26,27]. MIRU repeats are formed by a replicative mechanism confined to each individual locus [27]. Recently MIRUs have been used for typing of various bacterial species, including *Staphylococcus aureus*, *Bacillus anthracis*, different *Salmonella *and *Mycobacterium *species [9,24,27-34].

Within a population of a bacterial species, the variation in the number of copies of the repeat element at a specific locus indicates the diversity. VNTRs have been found in intergenic and nonintergenic regions of genomic DNA and have been found to function as molecular switches in microorganisms, by regulating transcription and possibly translation [29]. The exact stability of MIRUs of *M. avium *has not been studied. However MIRU-VNTRs are remarkably stable and therefore adequate for tracking key events in epidemiological investigations [22]. When used alone, this eight-locus-based typing system distinguished slightly fewer types of *M. avium *isolates than the IS*1245 *RFLP method. In our material most of the RFLP and MIRU clusters were congruent and the slightly lower discriminatory power of MIRUs is compensated by the better repeatability of the method. Further investigations are still needed before the wider application of MIRUs in mycobacterial epidemiological research.

In our study the major polymorphic site in both the human and porcine *M. avium *strain results was in locus TRX3. Therefore this locus has the highest discriminatory capacity in this material, suggesting that it may be highly sensitive for environmental variation and is in this sense the most informative one. In three loci (TR 3, TR 7 and TR 10) the same number of tandem repeats was found in all strains tested, suggesting that five MIRU loci could have the same discriminatory capacity as eight loci. In the future, our plan is to confirm this observation by studying a larger number of strains. The question also arises if certain genotypes or patterns are connected with a more virulent phenotype. The epidemiological significance of these similarities is unknown because data concerning epidemiological linkage between patients and pigs was not available. More isolates are needed to investigate the connection between different MIRU loci and virulence.

## Conclusion

In our setting RFLP typing and MIRU-VNTR typing provided a high level of both reproducibility and genetic diversity. The calculated DIs demonstrate that *M*. *avium *strains from different origins can be separated using RFLP typing or MIRU-VNTR typing method alone. The combination of the two typing methods confirms the relatedness of the strains. This was also shown in the study by Thibault et al. [9]. It has also been shown that the genetic variation between strains of *M. avium *subsp. *hominissuis *is generally higher than between *M. avium *subsp. *avium *strains [35]. *M. avium *subsp. *hominissuis *is usually found in human and porcine environmental mycobacterial infections [36]. The accuracy of the mycobacterial taxonomy and clinical significance could be increased by the application of several genetic tools for example the absence or presence of different genetic sequences [36].

So far, limited information is available about the utility of MIRU-VNTR typing to differentiate human and animal originating strains. Even small differences in MIRU-VNTR genotypes can be interpreted as evidence of the absence of a link, with a high degree of confidence [35]. MIRU-VNTR types of *M. avium *strains from environmental origins could clarify the role of tandem repeats and the infectiveness of the strains. In the future several MIRU loci that are linked to virulence and epidemiological traceability may be recognized. This may require a large amount of clinical field samples. In that case the MIRU-VNTR analysis may be applied in longitudinal and case control studies for epidemiological detection of potentially hazardous mycobacteria in humans and pigs.

## Competing interests

The authors declare that they have no competing interests.

## Authors' contributions

TT, TAV and JM participated in the discussion on the study design, collection of the samples and carried out the analysis. TT, TAV, JM, JP, ER, HM and OP participated interpretation of the data. TAV, JM, JP, ER, HM and OP helped to draft the the manucript. TT wrote the final manuscript. All authors read and approved the final manuscript.
